# Correction

**DOI:** 10.1080/19490976.2025.2457204

**Published:** 2025-01-24

**Article title**: Gut microbes with the gbu genes determine TMAO production from L-carnitine intake and serve as a biomarker for precision nutrition.

**Authors**: Wu, W. K., Lo, Y. L., Chiu, J. Y., Hsu, C. L., Lo, I. H., Panyod, S., … Wu, M. S.

**Journal**: *Gut Microbes*

**DOI**: https://doi.org/10.1080/19490976.2024.2446374

The figure 6 was published without the indicators which has been corrected in the original version and republished.

**Figure 6.**
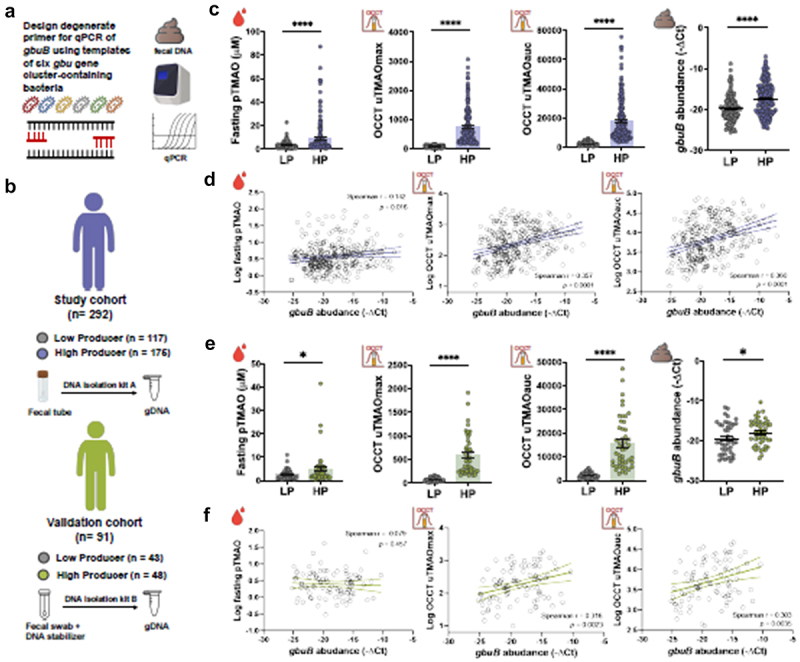
Figure 6. Development of a qPCR-based approach to quantify fecal gbuB gene abundance. (a) A pair of degenerate primer for amplifying gbuB was designed based on reference gbu-gene cluster of six gbu-containing bacterial species, including three type strains: Emergencia timonensis SN18, Eubacterium minutum ATCC700079, Agathobaculum desmolans ATCC 43058, and three new isolates: JAGTTR01 sp. NTUH-002-81, Agathobaculum sp. NTUH-O15-33, Intestinibacillus sp. NTUH-41-i26. (b) A study cohort (n = 292) and an external validation cohort (n = 91) were used to verify the qPCR-based fecal gbuB quantification approach. (c) The fecal gbuB abundance, as determined by qPCR, is significantly higher in high-TMAO producers than in low producers. (d) The fecal gbuB abundances determined by qPCR showed significant correlations with fasting plasma TMAO, OCCT uTMAOmax, and uTMAOauc. Among these, the latter two exhibited stronger correlation coefficients. (e) Using the same qPCR-based method, the abundance of fecal gbuB demonstrated a significant elevation in high-TMAO producers relative to low producers in an external cohort. (f) In the validation cohort, fecal gbuB abundances determined by the qPCR method exhibited significant correlations with OCCT uTMAOmax and uTMAOauc, but not with fasting plasma TMAO. Data were analyzed by using the unpaired Student t-test or Mann-Whitney U test as deemed appropriate. Data were presented as mean ± SEM. ****p < 0.0001, ***p < 0.001, **p < 0.01, *p < 0.05.

